# An epidemiological study of visceral leishmaniasis in North East Ethiopia using serological and leishmanin skin tests

**DOI:** 10.1371/journal.pone.0225083

**Published:** 2019-12-27

**Authors:** Desalegn Tadese, Asrat Hailu, Fitsum Bekele, Shewaye Belay

**Affiliations:** 1 Institute of Biomedical Science, College Health Science, Mekelle University, Mekele, Ethiopia; 2 Department of Microbiology, Immunology & Parasitology, Faculty of Medicine, Addis Ababa University, Addis Ababa, Ethiopia; 3 Department of Medical Laboratory Science, College of Medicine and Health Sciences, Wolkite University, Wolkite, Ethiopia; Universidade Federal da Bahia, BRAZIL

## Abstract

**Background:**

In Ethiopia, visceral leishmaniasis (VL) is caused by Leishmania donovani. The estimated country-wide incidence of VL in Ethiopia is 3700–7400 cases/year. The balance between anthroponotic and zoonotic transmission is still unknown even though most authors believe that visceral leishmaniasis in East Africa is anthroponotic. Asymptomatic leishmania infections occur more frequently than clinically apparent visceral leishmaniasis cases. The aim of this study was to determine the prevalence of asymptomatic VL infection and assess the degree of exposure among residents in Raya Azebo Woreda villages where cases of VL were recently reported.

**Methods:**

A community based cross-sectional survey was conducted in 2013 between 1^st^ of May and 25^th^ of July. A total of 1099 individuals living in 314 households were included in the study. Socio-demographic and clinical data were collected from each of the participants and venous blood was also collected for the detection of antibodies to visceral leishmaniasis using Direct Agglutination Test. Leishmanin skin test was performed to detect the exposure to the parasite. Data was entered into excel and exported to SPSS version 17 for statistical analysis. Chi-square and the corresponding p-values were used to determine the statistical significance of the proportions/ratios obtained from the cross tabulated data. A p-value *<* 0.05 was considered statistically significant.

**Result:**

A total of 1099 study subjects comprising 401 males and 698 females were included in the study. The overall positive leishmanian skin test and sero-prevalence rates respectively were 9.08% and 0.87%. The difference in LST positivity by age group and sero-prevalence by sex were statistically significant (P <0.01 and P<0.05 respectively). Out of the 9 sero-positive individuals, 7 had no history of travel to visceral leishmaniasis endemic areas out of Raya Azebo.

**Conclusion:**

In general our results suggest occurrence of VL in the study area is, very low. Our survey also indicates that due to the low incidence of the disease, and lack of awareness, some patients remain under diagnosed.

## Background

Visceral leishmaniasis (VL) is a protozoan parasitic disease caused by species of the Leishmania donovani complex [1]. Infection is achieved following a successful bite and inoculation of the infective stage, the promastigote, by the female phlebotomine sandfly [2].

According to the WHO estimates, about 500,000 new cases of VL occur every year globally [1]. 90% of which is borne by 6 countries: India, Bangladesh, Sudan, South Sudan, Brazil and Ethiopia [3]. In global estimates the highest number of VL cases are reported from South East Asia followed by; Sudan, South Sudan, Ethiopia, Kenya, and Somalia [4, 5]. East African region is among the areas where high number of VL cases are reported. Within the region, VL is prevalent in many foci in Eritrea, Ethiopia, Kenya, Somalia, Sudan, South Sudan and Uganda where the number of VL cases has increased markedly in the past decade [4, 6].

Ethiopia is the third most affected country, in the eastern African region, with an annual incidence of 3700–7400 cases [3]. The disease is known to be endemic in Metema and Humera plains in north west; in several localities of south western Ethiopia, i.e., the lower Omo plains, the Aba Roba focus in Segen valley, and Woito River valley adjacent to South Omo; in southern Ethiopia around Moyale area close to the borders with North Kenya; and in south eastern Ethiopia around Genale river basin in Oromia Regional State, Afder and Liban zones in Somali Regional State [4, 6, 7].Recent studies have also indicated that the disease is emerging in Benishangul Gumuz regional state in the west and Hamar and Banna -Tsamai districst of the Southern Ethiopia [7, 8].

Despite the fact that the disease is known to be endemic in the north west of the country, VL had not been a problem of the north east until recently. Increasing numbers of VL case reports from specific localities in some villages of Raya Azebo District, north east of the country, justifies the need to conduct this survey.

## Methods

### Study design

A community based cross-sectional survey was conducted in 2013 between 1st of May and 25th of July. The leishmanin skin test (LST) and Direct Agglutination Test (DAT) were employed to measure exposure to leishmania and to determine prevalence of asymptomatic infection.

### Study area

The study was conducted in Raya Azebo District of North Eastern Ethiopia. Raya Azebo is a District which is found in Southern Zone of Tigray, North East Ethiopia. According to the Ethiopian Central Statistics Agency (CSA) 2007 report, the District has a total population of 135,870 and has 13 rural and 3 urban kebelles (lower administrative unit in Ethiopia). The majority of the population (119,814) lives in rural kebelles. The main town of the District is Mehoni, which is located 651km away from Addis Ababa. The localities are situated in the lowland agro-ecological district, dominated by plains, undulating mountains and rugged terrain. The vegetation is mainly bush scrub and cactus with scattered acacia trees. Annual rainfall averages between 450 and 600mm. The annual temperature is between 16 and 26^oc^.

### Sample size

The sample size was determined by using web survey toolbox Version 1.04 software. There was dearth of data on VL prevalence for the study area. With the following assumptions:- the minimum prevalence of VL for serological test is 0.5%, 95% confidence interval, 5% degree of precision, 90% statistic al power and using 100% sensitivity/specificity test method, the sample size was calculated using simple binomial model. Accordingly, the final sample size calculated was: 1256

### Sampling sites and sampling technique

The sampling sites were selected in kebeles where VL cases were recently reported. Kebeles were identified by reviewing case records of Ayder referral Hospital. Based on the Hospital records, villages from Kara-Adisho and Bagea-Delewo kebeles were targeted. In these 2 kebeles, 4 villages/clusters were identified to comprise sampling sites based on a sample size of 1256 individuals. To include a study population of 1256 individuals, approximately 314 households were needed (average household size = 4). The 314 households were distributed across the 4 villages, proportional to the number of households in each village. Households were then randomly selected using the register that was available in the kebele office.

### Questionnaire and clinical examination

Socio-demographic characteristics were collected using pre-structured questionnaires. Past medical history, medical complaints and other demographic data was completed for every individual. A general clinical examination was performed in each individual with particular reference to hepatosplenomegaly, presence/absence of fever or recent history of fever, and presence of scars of previous cutaneous leishmaniasis and/ or post kala azar dermal leishmaniasis [PKDL].

### Sample collection and processing

Five ml of blood from adults and 3 ml from children was collected for serologic investigation. Serum was separated by centrifugation from coagulated blood, stored at-21 degree centigrade. LST was performed while in the field, whereas Direct Agglutination Test was performed in the facilities of Leishmaniasis Research and Diagnostic Laboratory-Addis Ababa University (LRDL-AAU).

### Leishmanin skin test

**Fig 1:**
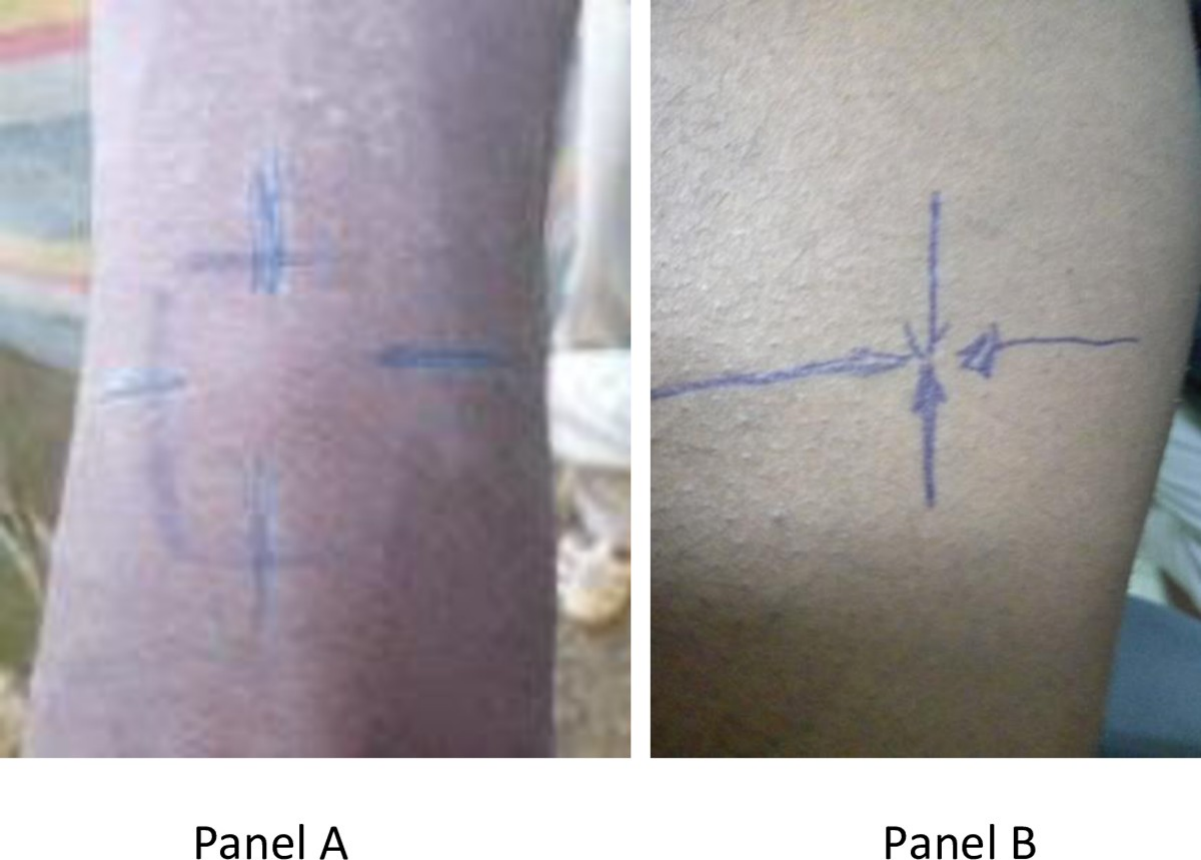
Positive (panel A) and Negative (panel B) LST reactions.

The leishmanin skin test (LST) is a test for Delayed Type Hypersensitivity (DTH) reaction specific to leishmania parasites, also known by the name Montenegro Test. The antigen (lot # 126-3/06-2010) prepared from L. major MRHO/IR/75/ER was injected on the volar aspect of the forearm. After 48 to 72 hours, the size of indurations was measured using the ball point method as previously described [9]. Results were recorded as average of two dimensional readings (Fig 1). Induration size of 5.0 mm and above were considered positive [9].

### Direct Agglutination Test (DAT)

**Fig 2:**
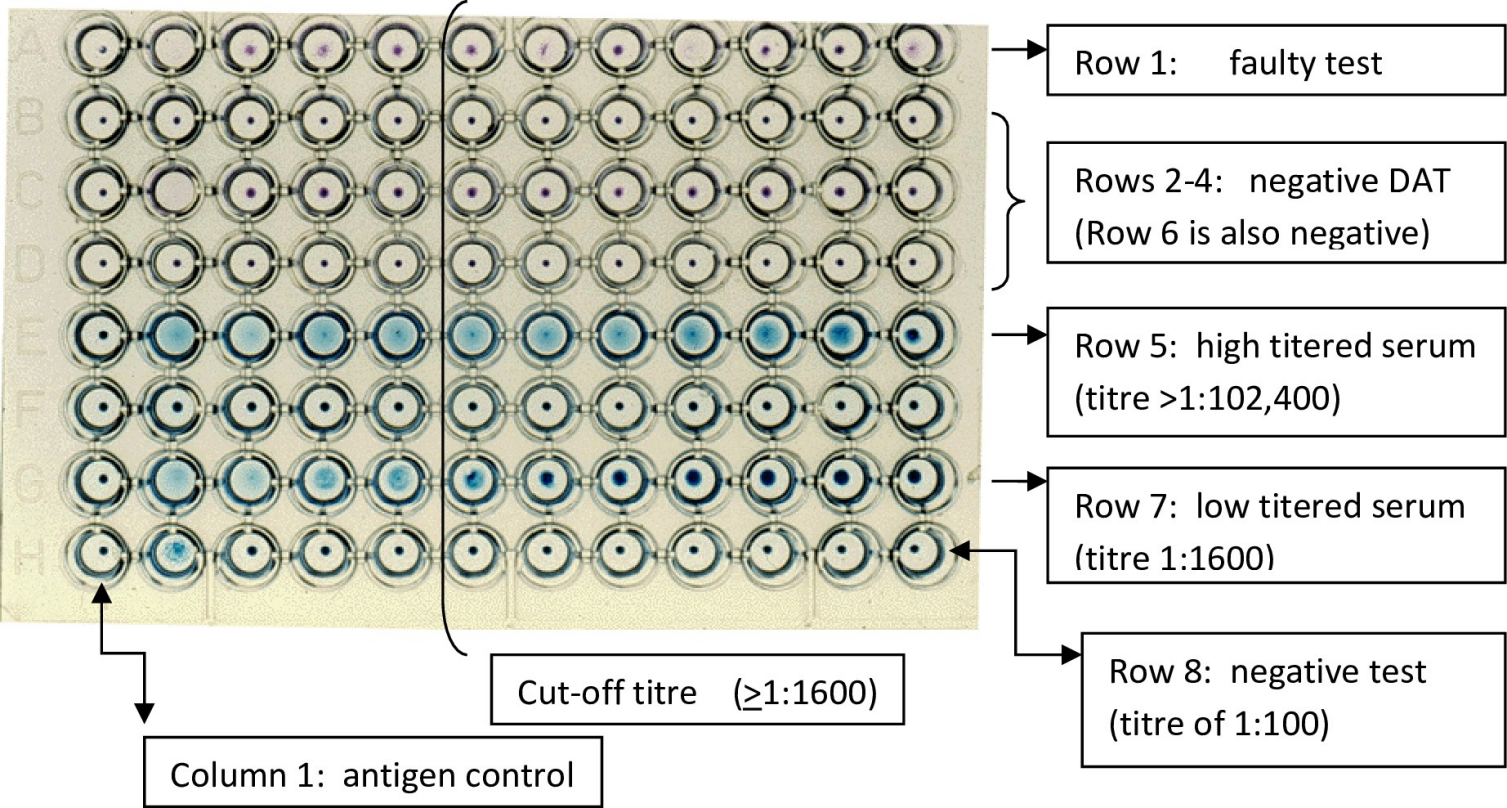
A plate showing DAT test results in sera tested with a starting dilution of 1:100 in column 2.

Serum was separated by centrifugation from coagulated blood. DAT was performed as previously described using a freeze-dried antigen obtained from KIT [10]. In brief, serially diluted serum samples were incubated in v-shaped micrtotitre plates with killed coomassie blue stained and fixed promastigotes of Leishmania donovani prepared from MHOM/SD/68/IS [10]. The plates were incubated at room temperature for 8–12 hours and then read visually. Agglutination was visible as a mat, or a dot with frayed edges or an enlarged dot [5, 10] as depicted in Fig 2.

### Data analysis

Data was entered into an excel sheet, checked for completeness, exported to SPSS version 20 and Epi Info 7, and analyzed by the same. Chi-square and the corresponding p-values were used to determine the statistical significance of the proportions/ratios obtained from the cross tabulated data. A p-value *<* 0.05 was considered statistically significant. Odds Ratio and the 95% CI were computed from a 2x2 table using Epi Info 7.

### Ethical clearance

Ethical clearance was obtained from "Addis Ababa University Research and Ethics Review Committee (meeting no 21; dated 16-01-2013, protocol no 03/M Parasitology). Before the recruitment, a detailed discussion was held with the participants, and with parents (or guardians of children). Written Consent form was provided to all participants. Participants were enrolled in the study after they understood the purpose of the study and after signing the informed consent form.

## Results

### Characteristics of the study population

A total of 1099 individuals; i.e., 700 (63.7%) females and 399 (36.3%) males greater than 2 years of age; were identified from 4 representative sites; Koban (38.9%), Tsaeda Meda (28.5%), Arada Mangudo (28.5%) and Delewo (6.3%) (Table 1). The male to female ratio was 1:1.75. The majority (42.7.0%) of the study subjects were in the age group 8–14 followed by; 0–7 years of age (16.7%). Those aged 36–42 were 4.4% of the study subjects.

**Table 1:** Sex distribution of study participants in different study sites, June 2013.

Study villages	Sex		Total
	Male	Female	
	# (%)	# (%)	# (%)
A/Mangudo	90(8.2%)	198(18.0%)	288(26.2%)
Koban	144(13.1%)	284(25.8%)	428(38.9%)
T/Meda	134(12.2%)	179(16.3%)	313(28.5%)
Delewo	31(2.8%)	39(3.5%)	70(6.3%)
Total	399(36.3%)	700(63.7%)	1099(100%)

### Leishmanin skin test (LST)

Leishmanin skin test was performed on 1099 study subjects, of whom 650 returned for reading. Among 650 study subjects who returned for LST reading: LST positivity rate showed variation by age groups in different geographic location (Table 2), gender (Table 3) and occupation (Table 4). The overall LST positive rate was 9.08% (11.77% % in males, 7.52%, in females) (S1 Data).

**Table 2:** Positive LST rates by age group shown by study sites, June 2013.

Age Group		Study sites			Total
	A/Mangudo	Koban	Tsaeda Meda	Delewo	
	#/n (%)	#/n (%)	#/n (%)	#/n (%)	#/n (%)
0–7	0/40(0.00%)	0/22(0.00%)	1/16(6.25%)	0/3(0.00%)	1/81(1.24%)
8–14	2/41(4.88%)	2/97(2.06%)	13/166(7.83%)	1/16(6.25%)	18/320(5.63%)
15–21	0/10(0.00%)	4/30(13.33%)	5/16(31.25%)	1/8(12.50%)	10/64(15.63%)
22–28	2/19(10.53%)	4/24(16.67%)	0/6(0.00%)	-	6/49(12.25%)
29–35	2/18(11.11%)	4/18(22.22%)	1/9(11.11%)	2/4(50.00%)	9/49(18.37%)
36–42	2/9(22.22%)	2/9(22.22%)	0/5(0.00%)	1/5(20.00%)	6/28(21.43%)
>42	4/23(17.39%)	3/18(16.67%)	2/12(16.67%)	1/6(16.67%)	10/59(16.95%)
Total	12/160(7.50%)	19/218(9.18%)	22/230(9.57%)	6/42(14.29%)	59/650(9.08%)

**Table 3:** LST rates shown by sex at different study sites, June 2013.

Village	Positive LST reading		Total
	Male	Female	
A/Mangudo	3/43(6.98%)	9/117(7.69%)	12/160(7.50%)
Koban	9/80(11.25%)	10/138(7.25%)	19/218(8.72%)
Tsaeda Meda	12/93(12.90%)	10/137(7.30%)	22/230(9.57%)
Delewo	4/22(18.18%)	2/20(10.00%)	6/42(14.29%)
Total	28/238(11.77%)	31/412(7.52%)	59/650(9.08%)

**Table 4:** LST rates shown by occupational groups and study localities, June 2013.

LST Positive					
Occupation	A/Mangudo	Koban	T/Meda	Delewo	Total
Governmental	0/5 (0.00%)	0/7 (0.00%)	0/5 (0.00%)	0 (0.00%)	0/17(0.00%)
Students	2/37 (5.41%)	2/97 (2.06%)	16/165(9.70%)	1/12 (8.33%)	21/311(6.75%)
Farmer	10/74(13.51%)	17/99(17.17%)	5/34 (14.71%)	4/21(19.05%)	36/228(15.79%)
Preschool	0/34 (0.00%)	0/11 (0.00%)	1/13 (7.69%)	0/5(0.00%)	1/63 (1.59%)
Total	12/150(8.00%)	19/214(8.88%)	22/217(10.14%)	6/38(7.90%)	58/619(9.37%)

Variations in the prevalence of leishmanin positivity were noted among the different study sites (Table 3). However, the difference in leishmanin positivity by sampling sites was not statistically significant (x^2^ = 5.28; P<0.508)

Variations in the prevalence of LST positivity were noted among the different occupations (Table 4). Farmers had the highest prevalence (15.79%), followed by students (6.75%), while the least prevalence was in pre-school (1.59%). The difference in LST by occupation was statistically significant (x^2^ = 25.43; P<0.05) (Table 4).

### Direct Agglutination Test (DAT) results

Of 1040 study participants, only one patient with clinical signs and symptoms consistent with VL, i.e., having fever, splenomegaly and anaemia was found. This patient was positive for both rK39-ICT and DAT at the time of the survey. In total, 9 individuals were DAT-positive; of which two had suffered kala-azar previously. The overall prevalence of infection (DAT- positive) was 0.87%. The prevalence among males was higher (1.87% versus 0.30%) (Table 5). Their difference was statistically significant (x^2^ = 7.27; P <0.05).

**Table 5:** Prevalence of anti-leishmanial antibodies measured by DAT shown by sex of the study participants in the different study sites, June 2013.

Village	DAT Positive		Total # (%)
	Male # (%)	Female # (%)	
A/Mangudo	2/77(2.60%)	2/182(1.10%)	4/259(1.54%)
Koban	0/136(0.00%)	0/274(0.00%)	0/410(0.00%)
Tsaeda Meda	1/131(0.76%)	0/172(0.00%)	1/303(0.33%)
Delewo	4/32(12.5%)	0/36(0.00%)	4/68(5.88%)
Total	7/376(1.86%)	2/664(0.30%)	9/1040(0.87%)

The highest prevalence was recorded in the age group 15–21 years (1.65%) and the 8–14 years age group showed the lowest prevalence (0.67%). The differences in DAT positivity rate by age group was statistically significant (x^2^ = 5.38; P<0.05) (Table 6).

**Table 6:** Sero-prevalence of anti-leishmanial antibody measured by DAT shown by age group and study sites, June 2013.

Village	Age group							Total # (%)
	0–7	8–14	15–21	22–28	29–35	36–42	>42	
A/Mangudo	1	1	1	1	0	0	0	4/ (1.54)
Koban	0	0	0	0	0	0	0	0/(0.00)
T/ Meda	0	1	0	0	0	0	0	1/(0.33)
Delewo	1	1	1	0	1	0	0	4 /(5.88)
Total	2/149	3/451	2/121	1/86	1/81	0/47	0/105	9/1040

Variations in the prevalence of DAT positivity were noted among the different study sites (Table 5). Delewo had the highest prevalence (5.88%) while the least prevalence was in Tsaeda Meda (0.33%), but there was not any DAT positive individual detected in Koban village. The difference in DAT positivity rates by study sites (village) was statistically significant (x^2^ = 34.90; P<0.05) (Table 5).

## Discussion

Microscopic examination of bone marrow, spleen, or lymph node aspirates to visualize parasite amastigotes has been the gold standard method of VL diagnosis for many years. However, because invasive sampling procedure and the expertise and facilities required, its use for asymptomatic infection surveillance is not justified.

The leishmanin skin test has been shown to be a valuable tool to measure exposure to leishmanial parasites particularly in epidemiologic surveys. On the contrary, lack of adequate sensitivity and specificity, and its ineffectiveness in distinguishing current from past exposure are among the drawbacks of this test. Nonetheless, its use in marking endemicity levels remains remarkable.

In this study, variation in LST positivity rates by age group and gender was observed. LST positivity rate varied from 1.24% in the age group 0–7 to 21.43% in 36–42 years old. It also increased steadily from a low positivity rate in early life to higher rates during adulthood, which is similar with many endemic regions of Ethiopia. The high proportion of LST positivity observed in the age group 15–21, 22–28 and in those greater than 42 years old in this study and the lower rate in those less than 7 years of age is in line with data from other studies carried out in endemic localities of Lower and middle Awash valley in Eastern Ethiopia and also Aba Roba focus in south western Ethiopia. [11, 12, 13]. The high proportion of positive LST in the age group above 15 is probably due to increased exposure to the insect bite because of outdoor activities.

In our study, variation in LST positivity was also observed among the different study sites (x^2^ = 5.28; P = 0.508). The highest LST positivity rate was documented in Delewo (14.29%), followed by Tsaeda Meda (9.57%), and Koban (8.72%). However, when compared with other studies carried out in endemic localities of the south, south central and south west Ethiopia it is low.[13, 14]. On the contrary, the overall prevalence of 9.08% which is observed in this study was relatively higher than another study carried out in Kenya and in the highland of northern Ethiopia [15, 16]. Marked ecological differences and different way of living the communities lead might have contributed for the observed differences.

The DAT is a semi qualitative test and highly adopted in field settings. The fact that it is simple to perform makes it ideal for both field and laboratory use. Its use especially for survey has been validated in several countries including India, Nepal, Bangladesh, Sudan, Ethiopia, Kenya, and Brazil [17].

In this study DAT positive rates varied from 0.00% to 5.88% among the different study villages. The overall DAT positivity was 9/1040 (0.87%), which was very low compared to findings in VL endemic areas of Ethiopia [7, 13, 18–20]. Majority of DAT positive study subjects (7out of 9) had no history of travel outside Raya Azebo Woreda, suggesting that the transmission took place in the study sites.

In this study differences, an increases in LST result and marked decreases in DAT, were observed. This might be due to the fact that the study area is one of the areas in the country which is highly endemic for cutaneous leishmanisis [21]. It is known that LST reading can be positive in other types of leishmnaiasis [22]. Apart from that, low number of study population who returned for LST reading might have also contributed.

## Conclusion

VL is a growing problem in Ethiopia. One way through which the burden could be reduced is by strengthening surveillance activities aimed at identifying asymptomatic/ subclinical infection. In VL Endemic countries in general and in Ethiopia in particular early identification and management of asymptomatic carriers has become a challenge for VL control programs.

In general our results suggest occurrence of VL in Our study area is, very low. Our survey also indicates that due to the low incidence of the disease, and lack of awareness, some patients remain under diagnosed. In-depth epidemiological/molecular and entomological studies are recommended.

## Supporting information

S1 Data Clinical and laboratory results of LST positive individuals at Raya Azebo 2013. (XLSX)
